# The brain structure and function abnormalities of migraineurs: A systematic review and neuroimaging meta-analysis

**DOI:** 10.3389/fneur.2022.1022793

**Published:** 2022-11-07

**Authors:** Zhu-Hong Chen, Yu-Ling Cui, Jing-Ting Sun, Yu-Ting Li, Chi Zhang, Yang-Ming Zhang, Ze-Yang Li, Yu-Xuan Shang, Min-Hua Ni, Bo Hu, Lin-Feng Yan, Wen Wang

**Affiliations:** ^1^Department of Radiology, Functional and Molecular Imaging Key Lab of Shaanxi Province, Tangdu Hospital, Fourth Military Medical University (Air Force Medical University), Xi'an, China; ^2^Department of Radiology, Gansu Hospital of Chinese Armed Police Force, Lanzhou, China; ^3^Department of Radiology, The First Affiliated Hospital, Xi'an Jiatong University, Xi'an, China; ^4^Department of Medical Technology, Shaanxi University of Chinese Medicine, Xianyang City, China

**Keywords:** migraine, magnetic resonance imaging, meta-analysis, systematic review, function, structure

## Abstract

**Objectives:**

To quantitatively summarize the specific changes in brain structure and function in migraine patients.

**Methods:**

A literature screening of migraine was conducted from inception to Sept 1, 2022, in PubMed, Web of Science, Cochrane Library, and Medline databases using the keyword combination of “migraine and MRI.” Activation likelihood estimation (ALE) was performed to assess the differentiation of functional connectivity (FC), regional homogeneity (ReHo), and gray matter volume (GMV) of migraine patients.

**Results:**

Eleven voxel-based morphometry (VBM) studies and 25 resting-state fMRI (rs-fMRI) studies (16 FC and 9 ReHo studies) were included in this study. ALE analysis revealed the ReHo increase in the brainstem and left thalamus, with no decreased area. Neither increased nor decreased regions were detected in FC and GMV of migraine patients.

**Conclusions:**

The left thalamus and brainstem were the significantly activated regions of migraine. It is a meaningful insights into the pathophysiology of migraine. The consistent alterated brain areas of morphometrical and functional in migraine patients were far from reached based on current studies.

## Introduction

Migraine is a complex neurological dysfunction characterized by recurrent attacks and pulsating headaches susceptible to physical or environmental factors. Broad clinical symptoms, such as nausea, vomiting, photophobia, and phonophobia etc., have been complained by suffers, with a headache duration ranging from 4 to 72 h (1). The estimated 1-year prevalence of migraine is about 15%, with a female-to-male ratio of 3:1 (2).

However, the underlying neuroimaging alterations in migraine patients have previously been studied using functional and structural MRI techniques, with inconsistent conclusions (3–5). Some studies reported the increased functional connectivity (FC) in prefrontal cortex, anterior cingulate cortex (ACC) (6), superior frontal gyrus, and temporal pole (7), while decreased FC in periaqueductal gray (PAG) (6), hypothalamus (8), ACC (9), temporal lobe (10), insular cortex (11) and amygdala (12). Among migraine patients, regional homogeneity (ReHo) was significantly increased in bilateral thalami, middle frontal gyrus and left insula (13), and decreased in putamen (14), cerebellum (15), and posterior cingulate cortex (PCC) (16). Meanwhile, voxel-based morphometry (VBM) studies suggested that brain gray matter volume (GMV) increased in PAG, bilateral fusiform gyri, and cingulate gyri (17), and decreased in cerebellar culmen (18), ACC, hippocampus (17), and orbitofrontal cortex (19).

If there are regions that both function and structure altered in migraine patients. Based on ReHo, amplitude low-frequency fluctuation (ALFF) and positron emission tomography (PET), meta-analysis demonstrated decreased activity in the angular gyrus, visual cortex, and cerebellum, while increased in the caudate, thalamus, pons, and prefrontal cortex (20). On the other hand, GMV decrease in posterior insular-opercular regions, the bilateral prefrontal cortex, and the anterior cingulate cortex were revealed with AES-SDM (3, 5). It is frustrating that a consistent conclusion was not drawn. Meanwhile, there still a lacks meta-analysis on the brain FC alterations in migraine patients. As more studies on the brain structure and function alterations in migraine patients have been published, it is urgent to perform a meta-analysis to draw a comprehensive conclusion including functional and structural studies.

Therefore, we conduct the current neuroimaging meta-analysis on brain structure and function changes in migraine patients, with the hope of drawing a solid conclusion.

## Materials and methods

This study was registered on the PROSPERO (https://www.crd.york.ac.uk/PROSPERO/), with the registration number CRD42021257300.

### Search strategy

A systematic literature search was conducted in the database of PubMed, Web of Science, Cochrane Library, and MEDLINE from inception to Sept 2022, according to Preferred Reporting Items for Systematic reviews and Meta-Analysis (PRISMA) (21). The subject terms and keywords, (“migraine” OR “primary headache”) AND (“magnetic resonance imaging” OR “neuroimaging” OR “fMRI”) AND (“structure” OR “voxel-based morphometry” OR “morphometrical” OR “functional connectivity” OR “regional homogeneity” OR “function”), were used to identify candidate VBM and rs-fMRI studies. Then, manual screening was conducted in the references of the retrieved studies and reviews.

### Inclusion and exclusion criteria

Studies that meet the following criteria were eligible for inclusion in this meta-analysis: (1) migraine patients diagnosed according to the International Classification of Headache Disorders (ICHD) (1); (2) MRI studies employed morphometric approaches of VBM, or functional metrics of FC and ReHo; (3) seed-based FC to whole-brain compared patients with migraine with health controls (HC) group; (4) coordinates were reported in Montreal Neurological Institute space (MNI), or Talairach space, and (5) peer-reviewed. Multiple papers published by the same author were included following the criteria: including the largest number of participants, latest published ones, and reported coordinates underwent stringent correction.

**Figure 1:**
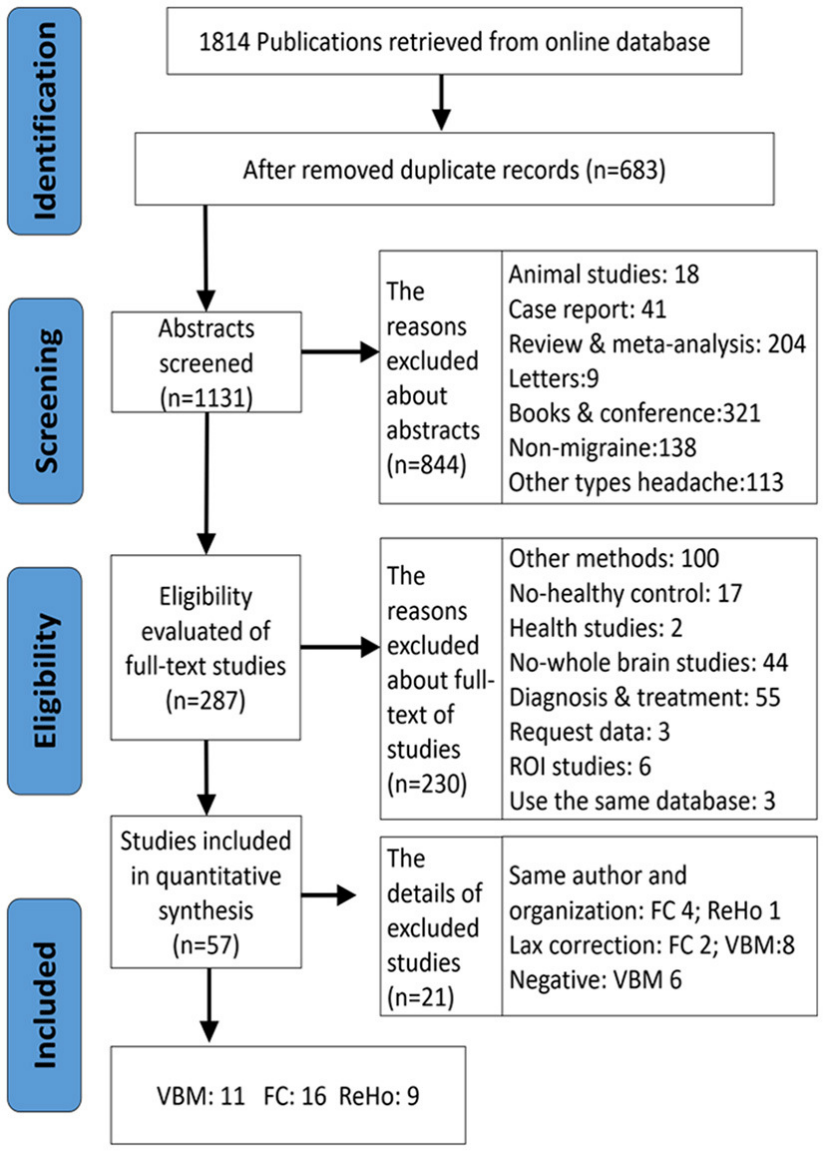
The flowchart of literature screening.

Exclusion criteria were as follows: (1) no HC group, (2) study was neither VBM nor FC and ReHo, (3) studies on the region of interest (ROI)-ROI, seed-ROI or independent component analysis (ICA), (4) intervention studies (pre/post-treatment contrasts such as transcranial magnetic stimulation or acupuncture), (5) seed-points or peak effect coordinates could not be retrieved, or (6) other types of migraine (e.g., vestibular migraine) and studies for comorbidities (Figure 1). We also excluded those studies that adopted lax statistical methods, like small volume correction (SVC) and uncorrected multiple comparisons.

### Data extraction

Articles retrieval, assessment, and data extraction were independently implemented by two authors (CZH and SJT) according to the data extraction protocol. Any vagueness or disagreements were discussed with a third author (CYL), and a consensus was reached. The data information was sequentially collected, such as author, published year, sample size, characteristics of participants (e.g., age, gender, disease duration, and attacks), classification of migraine, and technical details (MRI scanner, seed regions, and correction methods, etc.). The peak coordinates of included studies were edited as available files according to the guidelines of AES-SDM 5.15 (http://www.sdmproject.com/) (22) or ALE 3.02 (http://www.brainmap.org/) (23). Talairach coordinates were translated into MNI *via* a toolbox provided by Ginger ALE.

### Literature quality assessment

There is no consensus on the quality evaluation of neuroimaging studies up to now. We performed a customized checklist to assess the quality of included studies based on the assessment items of the Newcastle Ottawa Quality Assessment Scale (http://www.ohri.ca/programs/clinical_epidemiology/oxford.htm). The detailed items and scores of included studies are listed in Supplementary Tables S1, S2.

### Activation likelihood estimation (ALE) analysis

ALE evaluated the significant convergence between peak effect foci from different trials (e.g., migraine > controls, migraine < controls) for a given study in comparison with a random distribution of foci. It treated reported significant foci as spatial probability distributions centered on given coordinates rather than as single points. ALE assesses the cumulative probabilities of each voxel based on reported foci. ALE map was acquired after calculating the union probabilities of each voxel. The true convergence of foci and random clustering was tested by permutation tests. Based on the sample size and random effect model, the likelihood of consensus among different experiments is attained. Each focus is modeled as the center of a Gaussian probability distribution. Then, the modeled activation (MA) map for each study is generated. We employed the recommendation setting of cluster-level family-wise error (FWE) (*p* < 0.05) to carry out multiple comparisons, using an initial cluster threshold of uncorrected *p* < 0.001, and permutation tests were 5,000 (23).

### Sensitivity analysis

To assess the reliability and replicability of main resutls, we conducted a jackknife sensitivity analysis. The method was to repeat the process of removing one study and performing the others with the same meta-analysis at same threshold. If the main results remains significant in all or most of the combinations of the analysis, then it was regarded as rigorous.

### Subgroup analysis

Subgroup analyses were performed to evaluate the consistency of findings and to eliminate latent factors affecting main results. We conducted subgroup analysis of patients with migraine without aura to exclud clinical and methodological heterogeniety.

## Result

Thirty-nine MRI studies were included in this analysis, covering 11 VBM (Table 1), 16 FC (Table 2), and 9 ReHo studies (Table 3). It was comprised of 1,355 migraine patients (314 males and 1,041 females) and 1,149 (305 males and 844 females) HCs. Among them, VBM studies recruited 430 migraine patients (120 males, 328 females), and 317 HCs (93 males, 224 females); ReHo studies enrolled 337 migraine patients (77 males, 260 females), and 288 HCs (69 males, 219 females); FC studies included 588 migraine patients (135 males, 453 females), and 544 HCs (143 males, 401 females). All included structural and functional studies were performed statistical analyses for age and sex of included patients and controls (*t*-test or ANOVA, *p* < 0.05), individually. There were no significant differences in age and ratio of gender between migraine and HC, when the data were independently assessed. The preprocessing of fMRI images in all studies was performed by several steps, such as slice timing, realigning, normalizing, regressing nuisance covariates, filtering, and smoothing.

**Table 1:** Demographic and clinical characteristics of migraine in the VBM studies.

**Authors (years)**	**Migraine types**	**Patients**		**Disease**	**Disease attacks**		**Health controls**	
		**M/F**	**Age**	**Duration years**	**years**	**month**	**M/F**	**Age**
Neeb et al. (24)	EM	21 (6/15)	49.36 ± 7.62	26.71 ± 14.42	NA	5.33 ± 1.59	21 (6/1)	49.40 ± 7.79
	CM	21 (6/15)	49.04 ± 7.46	24.43 ± 8.3		17.38 ± 2.66		
Zhang et al. (16)	MWoA	32 (8/24)	38.3 ± 10.16	9.5 ± 6.23	NA	3.36 ± 2.55	32 (8/24)	38.8 ± 10.2
Bonanno et al. (25)	MWA	14 (0/14)	42.36 ± 2.95	5.21 ± 1.31	29.83 ± 11.9	NA	14 (0/14)	42.5 ± 5.17
	MWoA	14 (0/14)	43.5 ± 3.25	6.78 ± 3.66	22.75 ± 10.03			
Li et al. (26)	MWoA	72 (15/57)	21.3 (20.89; 21.73)	66.75 (32.19; 101.31) (month)	NA	5.89 (2.62; 9.16)	46 (12/34)	21.24 (20.98; 21.50)
Yu et al. (17)	EM	39 (9/30)	39.74 ± 11.59	NA	NA	3.75 ± 2.64	35 (15/20)	34.91 ± 10.89
	CM	17 (9/8)	49.59 ± 14.64					
Chen et al. (19)	EM	56 (19/37)	37.5 ± 7.6	194.6 ± 116.7 (month)	NA	13.8 ± 10.5	43 (15/28)	36.2 ± 7.7
	CM							
Chou et al. (27)	Migraine	40 (8/32)	39.2 ± 10.05	14.7 ± 10.2	NA	9.9 ± 6.5	27 (6/21)	41.3 ± 10.1
Masson et al. (4)	Migraine	19 (6/13)	32.7 ± 8.7	16.8 ± 7.4	NA	3.3 ± 1.1	19 (6/13)	33.6 ± 11.5
Hubbard et al. (28)	Migraine	17 (4/13)	41.71 ± 12.20	>3 (month)	NA	4-15	18 (4/14)	38.89 ± 11.25
Cao et al. (29)	MWoA	44 (11/33)	34.93 ± 10.66	10.34 ± 8.98	NA	10.14 ± 9.68	32 (16/16)	30.63 ± 9.56
Schading et al. (30)	Migraine	24 (1/23)	38.1 ± 12.5	20 ± 12.0	NA	5.7 ± 2.5	30 (5/25)	32.2 ± 10.3
**The technique details and main findings**.								
**Authors** (**years)**	**Diagnose criteria**	**Corrections**	**Scanners**	**Method**	**FWHM**	**Main findings**		
Neeb et al. (24)	ICHD-III beta	FWE	3.0T	VBM	10	Increased: right amygdala and right putamen, left putamen, right pallidum, right hippocampus, right PHG, right superior parietal lobule, left insula, right cerebellum, left superior occipital gyrus and cuneus		
						Decreased: frontal lobe, right angular gyrus.		
Zhang et al. (16)	ICHD-III beta	FDR	3.0T	VBM	8	Increased: bilateral cerebellar culmen, lingual gyrus, thalamus, fusiform and PHG		
Bonanno et al. (25)	IHS	FWE	3.0T	VBM	8	Increased: right superior parietal gyrus and left thalamus		
						Decreased: right cerebellum, left postcentral and precentral gyrus, right inferior frontal gyrus, and left lingual gyrus		
Li et al. (26)	ICHD-II	FWE	3.0T	VBM	8	Decreased: bilateral superior and inferior colliculus, PAG, LC, median raphe nuclei (MRN) and dorsal pons medulla		
Yu et al. (17)	ICHD-III beta	AlphaSim	3.0T	VBM	8	Increased: PAG dlPFC, left hippocampus/PHG		
						Decreased: ACC, bilateral dlPFC, left hippocampus/PHG		
Chen et al. (19)	ICHD-III beta	FWE	3.0T	VBM	8	Decreased: right orbitofrontal cortex		
Chou et al. (27)	ICHD-3	FWE	3.0T	VBM	8	Increased: left PCG		
						Decreased: right PCG, left precentral gyrus, and cerebellum		
Masson et al. (4)	NA	FWE	3.0T	VBM	15	Decreased: superior temporal areas and postcentral gyrus		
Hubbard et al. (28)	ICHD-II	GRF	3.0T	VBM	8	Increased: left hippocampus		
Cao et al. (29)	ICHD-III beta	FWE	3.0T	VBM	8	Decreased: middle frontal cortex		
Schading et al. (30)	ICHD-III	FWE	3.0T	VBM	3	Increased: left lingual gyrus		

ACC, anterior cingulate cortex; MWA, migraine with aura; MWoA, migraine without aura; EM, episodic migraine; CM, chronic migraine; FWHM, full width at half maximum; GRF, Gaussian random field theory; FEW, family wise error; FDR, false detect rate; SVC, small volume correction; mM, menstrual migraine; LF, low frequency; HF, high frequency; PAG, periaqueductal gray; LC, locus ceruleus; MRN, median raphe nuclei; dlPFC, bilateral dorsolateral prefrontal cortex; PHG, parahippocampal gyrus; ICHD-3 beta, international classification of headache disorders, 3rd edition (beta version); PCG, postcentral gyrus; HIS, international headache society; MTG, middle temporal gyrus; OFC, orbitofrontal cortex; T, Tesla; VBM, voxel-based morphometry.

**Table 2:** Demographic and clinical characteristics of FC studies in migraine.

**Author (years)**	**Migraine types**	**Patients**		**Disease**		**HC**	
		**Number (M/F)**	**Age (years)**	**Duration (years)**	**Attacks (month)**	**Number (M/F)**	**Age (years)**
Niddam et al. (31)	MwoA	26 (9/17)	32.3 ± 9.8	13.5 ± 8.0	2.6 ± 1.2	26 (9/17)	31.2 ± 5.8
	MWA	26 (9/17)	28.3 ± 7.5	13.1 ± 7.8	1.6 ± 1.0		
Zhang et al. (32)	MwoA	22 (9/13)	41.8 ± 10.2	9.8 ± 7.3	3.1 ± 2.2 ^#^	22 (9/13)	42.0 ± 10.3
Chen et al. (33)	EM	18 (4/14)	33.39 ± 10.69	12.44 ± 8.07	NA	18 (4/14)	39.11 ± 9.99
Li et al. (34)	MwoA	72 (15/57)	21.3 (20.89; 21.73)	NA	NA	46 (10/36)	21.24 (20.98;21.50)
Yu et al. (35)	MwoA	48 (11/37)	35.47 ± 9.91	9.38 ± 6.86	NA	48 (11/37)	35.12 ± 9.45
Meylakh et al. (36)	Migraine	26 (4/22)	30.6 ± 2.1	NA	NA	78 (12/66)	30.7 ± 1.3
Ke et al. (37)	MwoA	39 (9/30)	39.74 ± 11.59	NA	3.75 ± 2.64	35 (15/20)	34.91 ± 10.89
Meylakh et al. (38)	Migraine	34 (10/24)	32 ± 1.8	NA	NA	26 (4/22)	32.3 ± 2.3
Qin et al. (39)	MwoA	48 (14/34)	38.1 ± 10.4	8.5 ± 6.0	3.8 ± 3.3 ^#^	48 (14/34)	39.0 ± 11.0
Zhang et al. (40)	MwoA	30 (4/26)	39.87 ± 10.43	9.37 ± 7.77	5.17 ± 6.17	22 (8/14)	34.27 ± 8.34
Huang et al. (41)	MwoA	45 (12/33)	38.62 ± 10.11	13.8 ± 6.07	4.31 ± 4.34	40 (14/26)	35.45 ± 7.53
Wei et al. (42)	MWoA-A (27)	49 (7/42)	34.41 ± 9.75	7.11 ± 5.51	4.22 ± 2.19	20 (3/17)	33.4 ± 7.43
	MWoA-OA (22)		34.91 ± 12.14	7.50 ± 6.77	4.45 ± 3.00		
Cao et al. (43)	Migraine	30 (6/24)	36.1 ± 13.28	85.23 ± 54.78^*^	NA	40 (15/25)	36.88 ± 14.97
Gecse et al. (44)	MwoA	27 (6/21)	25.9 ± 4.6	NA	NA	27 (6/21)	25.6 ± 4.0
Gollion et al. (45)	MWA	21 (4/17)	39 (12)	25	15^*^	18 (5/13)	39 (9.5)
Yang et al. (46)	Migraine	27 (2/25)	34.89 9.070	11.11 ± 10.165	NA	30 (4/26)	35.53 ± 12.53
**Technique details and main findings of included FC studies**.								
**Author (years)**	**Seeds**	**Studies**	**MRI scanners**	**Diagnose criteria**	**Correction**	**Main findings**	
Niddam et al. (31)	MFG/AI/MPC	FC	3.0T	ICHD-II	FDR	Increased: left MFG, posterior cingulate and precuneus	
						Decreased: bilateral occipital lobes, right AI and basal ganglia	
Zhang et al. (32)	Precuneus/PCC	FC	3.0 T	ICHD-II	FDR	Decreased: left occipital gyrus, bilateral cuneus, bilateral parietal lobules, bilateral postcentral gyrus, bilateral dorsolateral prefrontal gyrus, pons, bilateral cerebellar posterior lobes, right paracentral lobule, right middle cingulate gyrus and bilateral SMA	
Chen et al. (33)	PAG	FC	3.0 T	ICHD-III beta	FDR	Decreased: left precentral gyrus, left MFG, left inferior parietal gyrus, bilateral middle temporal gyrus, right SFG, right SMA, right inferior frontal gyrus and medial SFG	
Li et al. (34)	Right precuneus	FC	3.0T	ICHD-II	FWE	Decreased: left precuneus, supramarginal gyrus and ITG	
Yu et al. (35)	Insulas	FC	3.0 T	ICHD-III beta	FWE	Increased: the frontal lobe, the caudate nucleus, and the THA	
						Decreased: temporal lobe, parietal lobe, cingulate gyrus, precuneus, PHG, and caudate nucleus	
Meylakh et al. (36)	PAG	FC	3.0T	ICHD-III beta	FDR	Increased: hypothalamus, THA	
Ke et al. (37)	Insula/cerebellum	FC	3.0 T	ICHD-III beta	FDR	Increased: bilateral SMA/PCL, right postcentral gyrus, left orbitofrontal gyrus and fusiform gyrus, bilateral temporal pole, and cerebellum	
						Decreased: bilateral angular gyrus, mPFC, hippocampus/PHG, middle/inferior temporal gyrus, left temporal pole, right cerebellum and brainstem	
Meylakh et al. (38)	Hypothalamic	FC	3.0T	ICHD-III beta	Bonferroni	Decreased: right hippocampus and bilateral ACC	
Qin et al. (39)	ADN/VPN	FC	3.0 T	ICHD-III beta	FWE	Decreased: left precuneus, right IPL and right MFG	
Zhang et al. (40)	LGN	FC	3.0T	ICHD-III beta	GRF	Increased: left cerebellum, right LG, left inferior frontal gyrus	
Huang et al. (41)	Amygdala	FC	3.0T	ICHD-III beta	GRF	Decreased: bilateral STG and right precentral gyrus	
Wei et al. (42)	LG	FC	3.0T	ICHD-III	Bonferroni	Increased: right PCC/precuneus, left MFG and left ITG	
Cao et al. (43)	Thalamus	FC	3.0 T	ICHD-III beta	FDR	Increased: left frontal gyrus	
Gecse et al. (44)	PAG	FC	3.0T	ICHD-III	FWE	Increased: cerebellum	
Gollion et al. (45)	Insula	FC	3.0T	ICHD-III	FDR	Increased: cerebellum	
Yang et al. (46)	Thalamus	FC	3.0T	ICHD-III	FWE	Decreased: precuneus, ACC, frontal gyrus	

# Times/month;

* The unit is month; CM, chronic migraine; EM, episodic migraine; MWA, migraine with aura; MWoA, migraine without aura; MWoA-A, migraine without aura with anxiety; MWoA-OA, migraine without aura and anxiety.

ACC: anterior cingulate cortex; AI: anterior insula; aMCC: anterior midcingulate; ADN: anterior dorsal thalamic nucleus; FC: functional connectivity; GMM: Gaussian Mixture Modeling; GMM: Gaussian Mixture Modeling GRF: Gaussian random field; IPL: inferior parietal lobule; IHS: International Headache Society; ITG: inferior temporal gyrus; LG: lingual gyrus; LGN: lateral geniculate nucleus; MCC: middle cingulum cortex; MFG: middle frontal gyrus; MOG: middle occipital gyrus; mPFC: medial prefrontal cortex; MPC: midline posterior cingulate; MRN: median raphe nuclei; MrD: marginal division of neostriatum; NA: not available; NCF: nucleus cuneiformis; OFC: orbitofrontal cortex; PAG: periaqueductal gray; PCC: posterior cingulate cortex; PCL: paracentral lobule; PHG: parahippocampal gyrus; RN: red nucleus; SFG: superior frontal gyrus; SMA: supplementary motor area; SN: substantia nigra; STG: superior temporal gyrus; THA: thalamus; VPN: ventral posterior nucleus.

**Table 3:** Demographic and clinical characteristics of migraine in the ReHo studies.

**Author (years)**	**Migraine types**	**Patients**		**Disease**		**Health control**	
		**Number (M/F)**	**Age**	**Duration (years)**	**Attacks (month)**	**Number (M/F)**	**Age**
Zhao et al. (13)	MWoA	Total:40 (12/28)	30.5 ± 10.8	10.15 ± 7.01^*^	NA	20 (5/15)	28.4 ± 8.9
		ST:20 (5/15)	27.12 ± 8.18	4.05 ± 1.64^*^	4.5 ± 3.5		
		LT:20 (7/13)	37.52 ± 10.2	16.25 ± 1.47^*^	5.38 ± 5.8		
Zhao et al. (14)	MWoA	19 (0/19)	21.8 ± 2.3	9.1 ± 2.6	NA	20 (0/20)	22.4 ± 3.1
Zhang et al. (47)	Migraine	MWoA:23 (6/17)	34 ± 8	9 ± 7	5.9 ± 9.4	25 (10/15)	35 ± 8
		MWA:12 (3/9)	32 ± 9	8 ± 6	2.5 ± 1.3		
Zhang et al. (48)	MWoA	30 (8/22)	41.0 ± 10.4	9.6 ± 6.83	3.3 ± 2.7	31 (9/22)	42.0 ± 10.3
Meylakh et al. (36)	Migraine	26 (4/22)	30.6 ± 2.1	14.56 ± 2.22	2.63 ± 0.64	78 (12/66)	30.7 ± 1.3
Chen et al. (49)	MWoA	IEM:19 (5/14)	42.0 ± 11.0	9.37 ± 3.62	1.56 ± 0.61	31 (13/18)	49.77 ± 13.69
		FEM:20 (4/16)	38.0 ± 12.01	9.80 ± 3.61	5.82 ± 2.06		
		CM:17 (9/8)	49.59 ± 14.64	7.41 ± 3.20	25.15 ± 6.87		
Li et al. (26)	MWoA	72 (15/57)	21.30 (20.89; 21.73)	66.75 (32.19–101.31)	5.89 (2.62–9.16)	46 (12/34)	21.21 (20.98;21.50)
Liu et al. (15)	MWoA	37 (6/31)	37.97 ± 9.82	16.19 ± 12.81	NA	15 (2/13)	34.88 ± 6.66
Lei and Zhang (50)	Migraine	22 (5/17)	33.32 ± 10.27	NA	NA	22 (6/16)	34.59 ± 7.99
**The technique details and main findings**.								
**Author (years)**	**Diagnose criteria**	**Study**	**MRI scanner**	**Corrections**	**Main findings**		
Zhao et al. (13)	IHS	ReHo	3.0T	FDR	Increased: bilateral thalamus, IFG, MOG, left insula, caudate, MFG, MTG, IOG, right ACC, MeFG, superior temporal gyrus, bilateral ACC, amygdala, thalamus, caudate, lentiform nucleus, uncus, SFG, temporal pole, cerebellum, brain stem, left hippocampus		
					Decreased: bilateral MFG, MTG, left lingual gyrus, right MOG, cerebellum, brain stem, bilateral ACC, insula, IFG, MFG, MeFG, SFG, MTG, MOG, cuneus, IPL, postcentral gyrus, precuneus, left fusiform gyrus, right PCC		
Zhao et al. (14)	IHS	ReHo	3.0T	FDR	Increased: thalamus, putamen, brainstem, cingulate cortex, inferior parietal gyrus hippocampus, OFC, and the occipital cortex		
					Decreased: putamen, brainstem, thalamus, temporal cortex, and cerebellum, OFC, secondary somatosensory cortex		
Zhang et al. (47)	ICHD-III beta	ReHo	3.0T	Alphasim	Increased: right occipital lobe		
					Decreased: right thalamus, right putamen, right frontal lobe, right hippocampus, right cerebellum, brainstem		
Zhang et al. (48)	ICHD-II	ReHo	3.0T	FWE	Decreased: bilateral S1 and the right PMC		
Meylakh et al. (5)	ICHD-III beta	ReHo	3.0T	FDR	Increased: PAG, hypothalamus, and somatosensory thalamus		
Chen et al. (49)	ICHD-III	ReHo	3.0T	FDR	Increased: bilateral thalami, right central anterior gyrus, left central posterior gyrus, right insular lobe, right sacral gyrus, bilateral central posterior gyri, right middle temporal gyrus, left olfactory cortex, right hippocampus, parahippocampal gyrus, suboccipital gyrus, cuneus, occipital gyrus		
					Decreased: bilateral prefrontal cortex, left angular gyrus, bilateral anterior cingulate cortex, prefrontal cortex, putamen and right supplementary motor area, bilateral prefrontal cortex, precuneus, putamen, anterior cingulate cortex		
Li et al. (26)	ICHD-II	ReHo	3.0T	FWE	Increased: bilateral MRN		
					Decreased: right middle occipital gyrus, inferior occipital gyrus,left middle occipital gyrus		
Liu et al. (15)	ICHD-III	ReHo	3.0T	FWE	Decreased: cerebellum		
Lei and Zhang (50)	ICHD-III	ReHo	3.0T	GRF	Increased: bilateral paracentral lobule		
					Decreased: bilateral ACC, cuneus, and lingual gyrus		

ST, short-term; LT, long-term. MWoA, migraine without aura; IEM, infrequent episodic migraine; FEM, frequent episodic migraine; CM, chronic migraine.

* Months.

ACC, anterior cingulate cortex; ICHD, international classification of headache disorders criteria; IFG, inferior frontal gyrus; HIS, international headache society; IOG, inferior occipital gyrus; IPL, inferior parietal lobule; MFG, middle frontal gyrus; MOG, middle occipital gyrus; MRN, median raphe nuclei; MTG, middle temporal gyrus; MeFG, medial frontal gyrus; OFC, orbitofrontal cortex; PCC, posterior cingulate cortex; PMC, premotor cortex; ReHo, regional homogeneity; S1, primary somatosensory cortex; SFG, superior frontal gyrus.

### Brain function alterations

**Figure 2:**
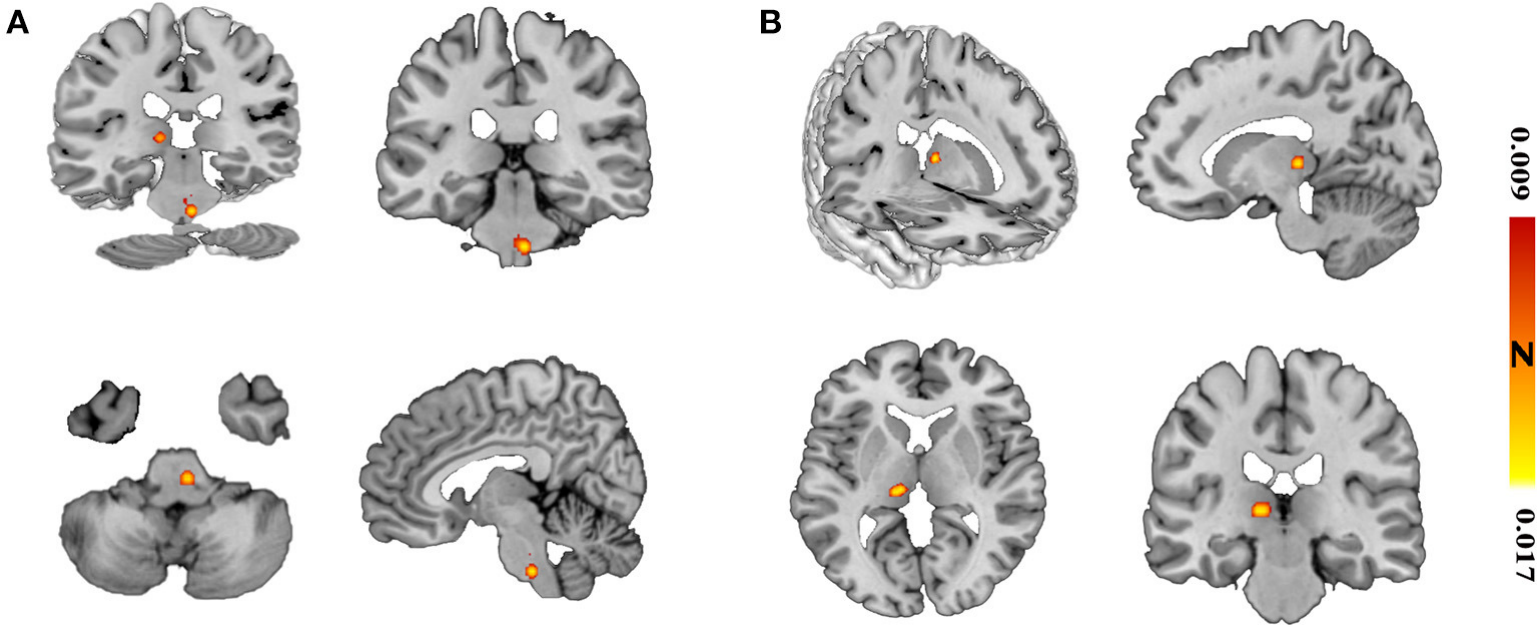
The spontaneous activity of ReHo in migraine patients. Using whole-brain overview and axial, sagittal, and coronal view, **(A)** illustrates the brainstem activating cluster (MNI: 6, −30, −44, 600 mm^3^); **(B)** shows the left thalamus activating cluster (MNI: −10, −24, 2, 560 mm^3^).

Using the coordinates of functional MRI studies to conduct ALE analysis, the ReHo values of left thalamus (MNI: −10, −24, 2; cluster volume 560 mm^3^) and brainstem (MNI: 6, −30, −44; 4, −28, −34; cluster volume 600 mm^3^) were increased, no decreased found. The changes of FC were not found (Figure 2, Table 4).

**Table 4:** The ReHo values changings of migraine patients by ALE analysis.

**Cluster**	**Volume (mm^3^)**	**Coordinate (MNI)**		**ALE value**	***z* values**	**Brain regions**
		***x***	***y***	***z***			
1	600	6	−30	−44	1.65 × 10^−2^	4.70	Brainstem
		4	−28	−34	0.98 × 10^−2^	3.61	
2	560	−10	−24	2	1.70 × 10^−2^	4.83	Left thalamus

MNI, montreal neurological institute space.

### Brain structure alterations

No VBM alterations were found in this analysis.

### Sensitivity analysis

ALE sensitivity analysis repeated the process of removing one study and performing the rest. We found that increased ReHo in brainstem and left thalamus was preserved throughout all studies, in spite of the most coordinates (80 foci) that reported by the Zhao's study (13) were not led to the instability of results (Table 5).

**Table 5:** Sensitivity analysis of ReHo in migraine patients by ALE software.

**Excluded studies**	**Volume (mm^3^)**	**Coordinate (MNI)**			**ALE value**	***z* values**	**Brain regions**
		***x***	***y***	***z***			
Zhao et al. (13)	752	−10	−24	2	1.70 × 10^−2^	5.24	Left thalamus
Zhao et al. (14)	608	6	−30	−44	1.65 × 10^−2^	4.72	Brainstem
	560	−10	−24	2	1.70 × 10^−2^	4.85	Left thalamus
Zhang et al. (47)	560	−10	−24	2	1.70 × 10^−2^	4.83	Left thalamus
Zhang et al. (18)	600	6	−30	−44	1.65 × 10^−2^	4.70	Brainstem
	560	−10	−24	2	1.70 × 10^−2^	4.83	Left thalamus
Meylakh et al. (36)	600	6	−30	−44	1.65 × 10^−2^	4.72	Brainstem
Chen et al. (6)	672	6	−30	−44	1.64 × 10^−2^	5.03	Brainstem
Li et al. (26)	592	6	−30	−44	1.65 × 10^−2^	4.71	Brainstem
	560	−10	−24	2	1.70 × 10^−2^	4.84	Left thalamus
Liu et al. (15)	600	6	−30	−44	1.65 × 10^−2^	4.68	Brainstem
	560	−10	−24	2	1.70 × 10^−2^	3.59	Left thalamus
Lei and Zhang (50)	600	6	−30	−44	1.65 × 10^−2^	4.70	Brainstem
	560	−10	−24	2	1.70 × 10^−2^	4.83	Left thalamus

### Subgroup analysis

According to the diagnosis classification of migraine, we performed subgroup meta-analysis of migraine patients and migraine without aura to establish the consistency of findings. No clusters were above the threshold.

## Discussion

To our knowledge, this is the first study adopting functional and structural fMRI metrics to verify brain alterations (VBM, FC, and ReHo) in migraine patients. The solid conclusion is that the ReHo values of left thalamus and brainstem were consistently increased. While GMV and FC were not illustrated alterations in migraine in terms of current evidence.

Migraine is associated with various central nervous system disorders (2). Profoundly prolonged duration and recurrently attacked headache are the main complaints of migraine suffers (51). Thalamus is thought to have an essential role in the pathophysiology of migraine and has been investigated extensively (52–55). Meanwhile, the migraine genesis is more likely within brainstem, involving dysfunction and plasticity changes (56).

In migraine patients, pain information is transmitted from the meninges to the brain *via* the trigeminovascular pathway starting from trigeminal ganglion neurons (57). Specifically, the spinal trigeminal nucleus (SpV) neurons convey nociceptive signals to the brainstem (such as periaqueductal gray, reticular formation), hypothalamic, and basal ganglia. Then, the relay thalamic neurons project to the somatosensory, insular, motor, parietal association, auditory, visual, and olfactory cortices to construct the specific properties of migraine pain (58), for instance, nausea, vomiting, lacrimation, anxiety, and hypothalamic-regulated functions like appetite loosing and fatigue (56).

ReHo is specialized in explore local connectivity in a specific region by characterizing its relationship with nearby voxels in a specific region (59). Meta-regression analysis has indicated that migraine patients' visual analog scale score was associated with increased brain activity in the left thalamus (20). Using different meta-analysis method and sensitivity analysis, we also concluded that the left thalamus and brainstem of migraine patients were more spontaneous activated than HCs. Based on these evidences, we speculate that left thalamus and brainstem maybe the biological markers of nociceptive information transmission in frequent migraine attacks.

We postulated that if there is a certain region affected by long-term migraine, the functional connectivity changes of cerebral regions could be convergent at ones regardless of the chosen of the seed-points. Totally, 16 FC studies were included in this study. Among them, the seed-points were distributed the middle frontal gyrus (31), precuneus (32), PAG (33, 36, 44), insulas (35, 37, 45), thalamus (38, 43, 46), pons (26, 39), lateral geniculate nucleus (40), amygdala (41), and lingual gyrus (42). Although, the regions exhibited FC alteration among those studies, involving cortex about pain processing, visual, auditory, affective, and cognitive evaluation, there is no solid conclusion of pain information projecting of migraine temporarily according to our analysis.

Meanwhiles, the GMV changes of migraine assessed by VBM were heterogeneous between previous studies and meta-analysis (3–5). Now, a tendentious consensus of no structural brain alterations is more acceptable by researchers (60, 61). Furthermore, after rigorous literature screening, no morphometrical changes were detected with meta-analysis using different software.

## Conclusion

The first quantitative coordinates meta-analysis of whole-brain neuroimaging studies for migraine that synthesized functional and structural MRI metrics, with the aim of providing the most comprehensive insights into brain impairments of migraine patients. Our meta-analysis suggested spontaneous cerebral activity in the left thalamus and brainstem, with no FC and GMV alterations. The findings may be served as the brain dysfunction clue of the underlying pathophysiology of migraine. In addition, neuroimaging meta-analysis, for reliable and robustness results, rigorous literature screening is prerequisite.

## Limitation

Firstly, the heterogeneity analysis, and correlation analysis were not carried out due to the ALE software restriction. The number of included studies was insufficient to perform subgroup analysis. Secondly, unpublished studies (“gray studies”) were not included in our meta-analysis, which inevitably leads to publication bias. And the coordinates-based meta-analysis also has inherently biased, as it employs pooled stereotactic coordinates that are statistically significantly different, rather than raw data. Thirdly, this meta-analysis was limited to seed-based to whole-brain fMRI studies of FC, the studies using independent component analysis (ICA) and positron emission tomography (PET) approach not included.

## Strengths and limitations of this study

- Functional and structural changes were evaluated simultaneously.
- Robust results were attributed to the rigorous processing analyses.
- More studies are needed to verify the changes in GMV.

## Author contributions

Z-HC, Y-LC, J-TS, Y-TL, and CZ devoted to this study equally as the co-first authors. Z-HC wrote the original draft. Y-MZ, Z-YL, Y-XS, and M-HN were constrictive for data abstraction and software analysis for this study. BH and L-FY monitored the analysis procedure. WW supervised the overall procedure. All authors revised and approved the final manuscript.

## Funding

This work was supported by the Hovering Program of Fourth Military Medical University (axjhww to WW), and the Talent Foundation of Tangdu Hospital (2018BJ003 to WW).

## Conflict of interest

The authors declare that the research was conducted in the absence of any commercial or financial relationships that could be construed as a potential conflict of interest.

## Publisher's note

All claims expressed in this article are solely those of the authors and do not necessarily represent those of their affiliated organizations, or those of the publisher, the editors and the reviewers. Any product that may be evaluated in this article, or claim that may be made by its manufacturer, is not guaranteed or endorsed by the publisher.

## Abbreviations

ACC: anterior cingulate cortex
ALE: activation likelihood estimation
BA: brodmann area
CM: chronic migraine
DMN: default mode network
FC: functional connectivity
FEW: family wise error correction
GMV: gray matter volume
HC: healthy controls
ICA: independent components analysis
ICHD: international classification of headache disorders
HIS: international headache society
MA: modeled activation
MNI: montreal neurological institute space
MWA: migraine with aura
MWoA: migraine without aura
PAG: periaqueductal gray
PCC: posterior cingulate cortex
PHG: parahippocampal gyrus
PRISMA: preferred reporting items for systematic reviews and meta-analysis
ReHo: regional homogeneity
ROI: region of interest
rs-fMRI: resting-state fMRI
SpV: spinal trigeminal nucleus
SVC: small volume correction
VBM: voxel-based morphometry.
