# Supplementary material for: The brain structure and function abnormalities of migraineurs: A systematic review and neuroimaging meta-analysis
Source: Front Neurol. 2022 Nov 7;13:1022793. doi: 10.3389/fneur.2022.1022793 (PMC9676357; doi:10.3389/fneur.2022.1022793)
Supplement: Supplementary file 1 [file Table_1.doc]

**Table 5S.** Quality assessment checklist.

**Subjects:**

1. Migraine was diagnosed according to the IHS criteria. Demographic data of patients were reported.

2. Healthy controls with medication overuse, psychiatric illness, and other types of headaches were excluded.

3. Age, gender, disease duration, attacks, and comorbidity were reported.

4. Patients with migraine and healthy controls came from the same general population.

5. The sample size of patients with migraine and healthy controls was more than 10.

**Imaging data acquisition and analysis:**

6. MRI field strength ≥ 1.5T.

7. The rs-fMRI studies were analyzed as FC between a seed point and the whole-brain. ROI and a priori regional analysis were excluded.

8. The procedure of imaging data processing and the software were clearly described.

9. Significant coordinates were reported in standard stereotactic space.

**Results and conclusions:**

10. Adopted recommended statistics and corrected for multiple comparisons (FWE/FDR/AlphaSim) to achieve significance convergence.

11. The parameters (*p*, *t*, or *z*) of resulting coordinates were indicated.

12. The conclusion was consistent with the results. The limitations were discussed.

Each item was scored as a 1 or 0 if it fully or partially fitted the bill, respectively. IHS: International headache society; FC: functional connectivity; ROI: region of interest; FWE: family-wise error; FDR: false discovery rate; T: tesla; rs-fMRI: resting-state functional magnetic resonance imaging.
